# Discharge-delaying factors for patients suitable for outpatient parenteral antimicrobial therapy (OPAT) in an Irish tertiary hospital during COVID-19

**DOI:** 10.1093/jacamr/dlab163

**Published:** 2021-10-14

**Authors:** S Staunton, D Rajendran, P Maher, J McCann, S McNicholas, E Feeney, C O’Broin, S Savinelli, P Mallon, A Watson, S Waqas

**Affiliations:** 1 Department of Medicine, St Vincent’s University Hospital, Elm Park, Dublin 4, Ireland; 2 Department of Infectious Diseases, St Vincent’s University Hospital, Elm Park, Dublin 4, Ireland; 3 Department of Radiology, St Vincent’s University Hospital, Elm Park, Dublin 4, Ireland; 4 Department of Microbiology, St Vincent’s University Hospital, Elm Park, Dublin 4, Ireland; 5 UCD School of Medicine, University College Dublin, Belfield, Dublin 4, Ireland

Outpatient parenteral antimicrobial therapy (OPAT) enables the outpatient treatment of patients requiring intravenous (IV) antibiotics who are clinically stable enough to safely receive this treatment at home.^1^ It facilitates earlier discharge and sometimes admission avoidance of patients and is an important antimicrobial stewardship tool. Delays to the discharge of OPAT-suitable patients can add to unnecessary hospital bed occupancy. This has gained particular relevance during the current COVID-19 pandemic which has caused increased demand for beds and risk of nosocomial COVID-19 acquisition.^2^ We recently conducted a study to assess factors delaying the discharge of hospital patients to OPAT services.

Fifty-three patients were referred for OPAT during the 3 month study from 8 October 2020 to 8 January 2021. Most of the referred patients were suitable for and discharged to OPAT services (*n = *37, 70%). In line with international standards,^3^ delayed discharge from hospital was defined as discharge occurring >24 h after the patient was formally declared suitable for OPAT. More than half of patients experienced delays to their discharge (*n = *21, 57%). The mean time from being declared suitable for OPAT to discharge was 2.8 days (range 0–11). A lack of capacity in community OPAT services was the most frequent factor delaying discharge (*n = *10, 48%), followed by a delay in peripherally inserted central catheter (PICC) insertion (*n = *8, 38%). PICC was the IV access of choice for 95% (*n = *35) of patients. Mean time from PICC request to insertion was 4.2 days (range 0–12). The time to PICC insertion was longer than the time to discharge, as many patients had their PICCs requested prior to being referred for OPAT during their acute presentation. OPAT PICCs are prioritized in our institute, but the term ‘OPAT’ was included in less than half of all requests for OPAT PICCs (*n = *14, 45%). Three patients (10%) were delayed as they were unavailable for transfer when a porter arrived to bring them for PICC insertion.

Delayed discharge was of particular interest as this study was conducted during the COVID-19 pandemic. During the data collection period, 57% of patients experienced delayed discharges, a figure which is a clear worsening relative to previous audits conducted in the pre-COVID era. Locally available data indicates that 36%–39% of patients experienced delays in discharge to OPAT in 2018 and 2019, demonstrating the impact the pandemic had on the OPAT services.

Eighty-nine additional bed days were attributed to these delays, with an estimated cost of €76 095. This indicated inefficient use of resources. In an attempt to reduce the delay to discharge for OPAT-suitable patients highlighted by our study, targeted communication was made to all interns in our hospital requesting that the word ‘OPAT’ be included in all PICC requests for OPAT to assist radiology in the appropriate prioritization of these requests and that this would be communicated again to the new cohort of interns when they start (usually July each year). We have suggested that radiology contact the ward prior to sending a porter to confirm patients are ready in order to minimize inefficiencies due to patients being unavailable for their slot. Midline catheters (MCs) are an alternative form of IV access to PICCs. The safety of MCs for OPAT duration longer than 2 weeks is not well established, and international guidelines currently recommend PICCs over MCs for OPAT where longer than 2 weeks IV access is anticipated.^4^ The majority (73%, *n = *27) of the patients in our institution receive OPAT for longer than 2 weeks due to a preponderance of vascular and orthopaedic infections. For these reasons, PICC remains the access of choice for OPAT at our institution. Findings from this study have been presented at national microbiology and infectious disease specialty meetings to highlight the need to increase the capacity for community OPAT. We plan to repeat this study in due course to assess the effects of these interventions.

In conclusion, many patients experienced delays in discharge to OPAT services during our study period despite being suitable for OPAT. Studies to look at discharge-delaying factors for OPAT-suitable patients similar to this study should be repeated in other institutions to optimize procedures and maximize the benefits from OPAT.

## Funding

This study was carried out as part of our routine work.

## Transparency declarations

None to declare.
